# A Vascular Intervention Assist Device Using Bi-Motional Roller Cartridge Structure and Clinical Evaluation

**DOI:** 10.3390/bios11090329

**Published:** 2021-09-10

**Authors:** Jueun Choi, Sangeun Park, Young-Hak Kim, Youngjin Moon, Jaesoon Choi

**Affiliations:** 1 Department of Biomedical Engineering, Asan Medical Center, University of Ulsan College of Medicine, Seoul 05505, Korea; cje20205392@mail.ulsan.ac.kr; 2 Biomedical Engineering Research Center, Asan Institute for Life Sciences, Asan Medical Center, Seoul 05505, Korea; pse0216@amc.seoul.kr; 3 Department of Cardiology, Asan Medical Center, University of Ulsan College of Medicine, Seoul 05505, Korea; mdyhkim@amc.seoul.kr; 4 Department of Convergence Medicine, Asan Medical Center, University of Ulsan College of Medicine, Seoul 05505, Korea

**Keywords:** cardiovascular intervention, medical robot, teleoperation control, master–slave system, guidewire

## Abstract

Conventional vascular intervention procedures present issues including X-ray exposure during operation, and an experience-dependent success rate and clinical outcome. This paper presents a novel robotic system using modularized bi-motional roller cartridge assemblies for robotic vascular interventions, specifically percutaneous coronary interventions (PCIs). The patient-side robot manipulates instruments such as the guiding catheter, guidewire, balloon/stent catheter, and diagnostic sensor catheter via commands from the user interface device, which is controlled by the physician. The proposed roller cartridge assembly can accommodate instruments of various sizes with an active clamping mechanism, and implements simultaneous translation and rotation motions. It also implements force feedback in the physician-side system, to effectively monitor the patient-side system’s status. The positioning accuracy and precision in using the robotic system showed satisfactory performance in a phantom-based test. It was also confirmed, through animal experiments and a pilot clinical trial, that the system demonstrates feasibility for clinical use.

## 1. Introduction

Percutaneous coronary intervention (PCI) is one of the representative vascular intervention procedures used to treat coronary artery disease (CAD) in a minimally invasive manner. However, the procedure has an unavoidable dependence on fluoroscopy or angiography systems [1,2], and the clinical outcome and complication rate vary widely among physicians with different experience levels. Long, flexible instruments—catheters or guidewires—are inserted by the physician through a vascular tree in the beating heart, with inevitable nonlinearity and complexity in controlling the motion, requiring long-term training. Even for experienced physicians, a considerably long time may be needed, depending on the type of procedure [3,4]. Robotic systems, to assist cardiovascular and peripheral vascular intervention procedures, have been developed. The robotic systems’ primary advantage is the reduction in radiation exposure, due to the teleoperation configuration [5,6,7]. Additional advantages may include increased precision, accuracy, and ease of motion of vascular instruments. Numerous research groups are continuously presenting new research and development attempts, and the interest of physicians in robotics technologies has been significantly increasing.

Mahmud et al. [8] provided a review on robotic technology in interventional cardiology. Commercial products, including Magellan^TM^ (Hansen Medical, Mountain View, CA, USA) [9,10,11] and CorPath^®^ (Siemens Healthineers, Erlangen, Germany) [12], have received approval from the United States Food and Drug Administration. Magellan^TM^ uses proprietary robotic catheters, while CorPath series robots provide only teleoperation functions for conventional catheters and guidewires [8]. Furthermore, neither of the above solutions include haptic or force feedback functions in the master device of the teleoperation system [13]. In the research domain, Thakur et al. presented a master–slave catheter navigation system, with two degrees of freedom (DOF), and translational and rotational motions [14,15,16]. Lee et al. [17] have also developed a robotic system designed explicitly for interventional radiology procedures, such as transcatheter arterial chemo-embolization (TACE), which may have limitations in cardiovascular procedure application.

In this paper, a novel design of a robotic vascular intervention system is presented. When compared with the existing systems, our robotic system has the following features. Our system enables simultaneous control of the vascular instrument’s translational and rotational movements, through a unique bi-motional modular roller cartridge mechanism. Compared to the individual translational and rotational motions, the simultaneous motion enables more effective navigation of the guidewire to the desired branch [18,19]. The proposed modularized robotic system enables quick setting and retrieval of the vascular instruments in an emergency response situation. The active clamping mechanism allows the use of various vascular instruments with different diameters and internal structures, such as guiding catheter extension and intra-vascular ultra-sound (IVUS) catheters. Finally, the master control device, designed as a haptic device, can aid in the physician’s intuitive operation of the system with augmented information. 

## 2. Methods

### 2.1. System Concept and Design

#### 2.1.1. System Concept

**Figure 1:**
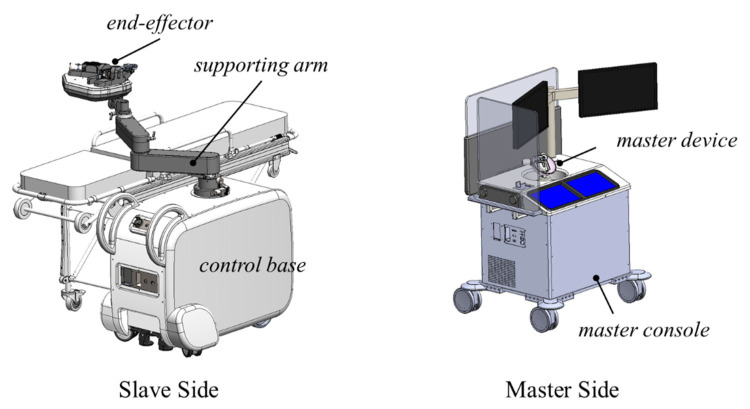
Overall configuration of the system.

As shown in Figure 1, our robotic system has a master–slave-type teleoperation configuration that allows the physician to avoid X-ray exposure. The master side is operated by the physician, while the slave side is used for the operation on the patient [20,21]. The end-effector of the slave-side system features a set of roller cartridges that can perform both translation and rotation and accommodate instruments with various diameters by an active clamping mechanism in one unified unit for modular configuration. 

The translation mechanism advances or retracts vascular instruments by axially rotating the tightly pressed roller pairs. The rotation mechanism rotates vascular instruments by rubbing the roller pair. The roller mechanism was chosen for uninterrupted translational motion control of the vascular instruments [22,23,24].

#### 2.1.2. Design of Slave End-Effector

**Figure 2:**
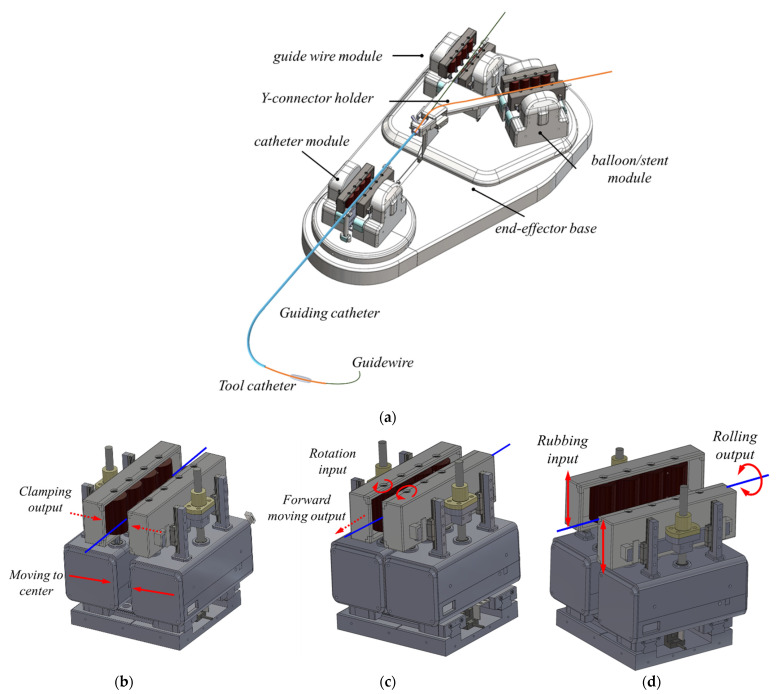
(**a**) The slave end-effector and the motion principles of (**b**) clamping mechanism, (**c**) translation mechanism, and (**d**) rotation mechanism in the roller module.

Based on this concept, the end-effector in the slave robot shown in Figure 2a consists of three modules for three types of instruments, a y-connector supporter, and tool supporters. Three modules are used to separately actuate a guiding catheter, guidewire, and balloon catheter. The guiding catheter and guidewire module are 3-DOF roller systems that clamp, translate, and rotate a target tool shown in Figure 2b–d. The balloon module is a 2-DOF roller system identical to the other modules, but does not have a rotation function. The guidewire and balloon catheter modules are mounted on a movable base plate in order to maintain the distance to the catheter module, depending on the guiding catheter’s translation.

**Figure 3:**
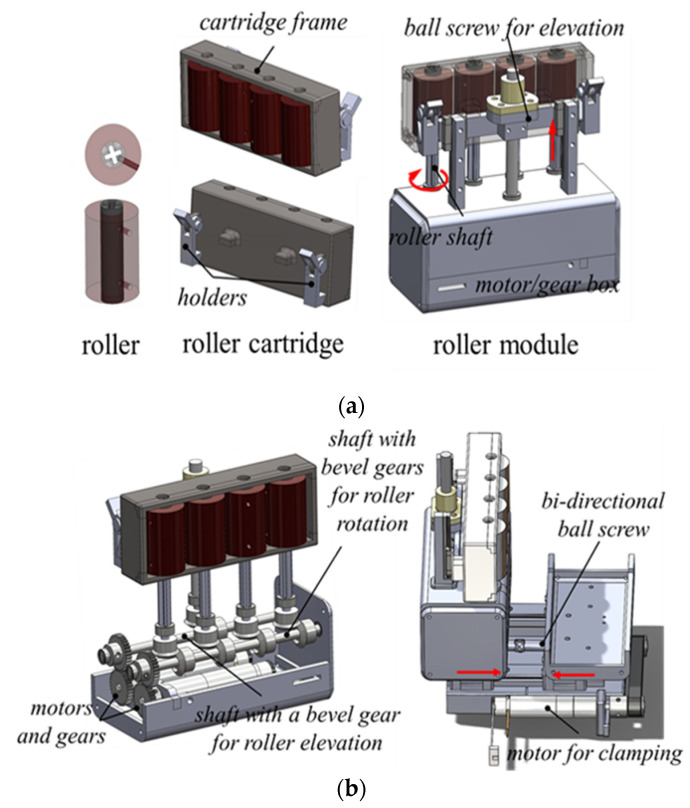
(**a**) Design of roller, roller cartridge, and side compartment of roller module. (**b**) Design of motor/gear box and clamping device of roller cartridge.

The roller module’s side compartment consists of a motor/gearbox and cartridge frame with four rollers, as shown in Figure 3. A roller with a 20 mm diameter has a cross-shaped hole for easy insertion into a roller shaft on the motor/gearbox. A roller cartridge contains four rollers and a holder attached to the back of the cartridge frame, allowing it to be quickly assembled and disassembled with the motor/gearbox. The roller cartridge is designed as a disposable part. In the motor/gearbox, there are motors for translation and rotation, and a gear system. Four cross-shaped roller shafts and two linear motion (LM) guide blocks with a horizontal bar are vertically attached to the motor/gear box. The rollers and the holders on the cartridge frames are assembled with the roller shafts and the horizontal bar, respectively. In Figure 3, a motor is connected to a shaft with bevel gears for roller rotation for the translation mechanism. For the rotation mechanism, a motor is connected to another shaft with a bevel gear for roller elevation, and the cartridge frame assembled with the bar is elevated during rotation. For the clamping mechanism, a clamping motor is connected to a bidirectional ball screw by a timing belt, and the distance between the two compartments attached to each LM guide can be adjusted while clamping.

#### 2.1.3. Design of Master Haptic Device

**Figure 4:**
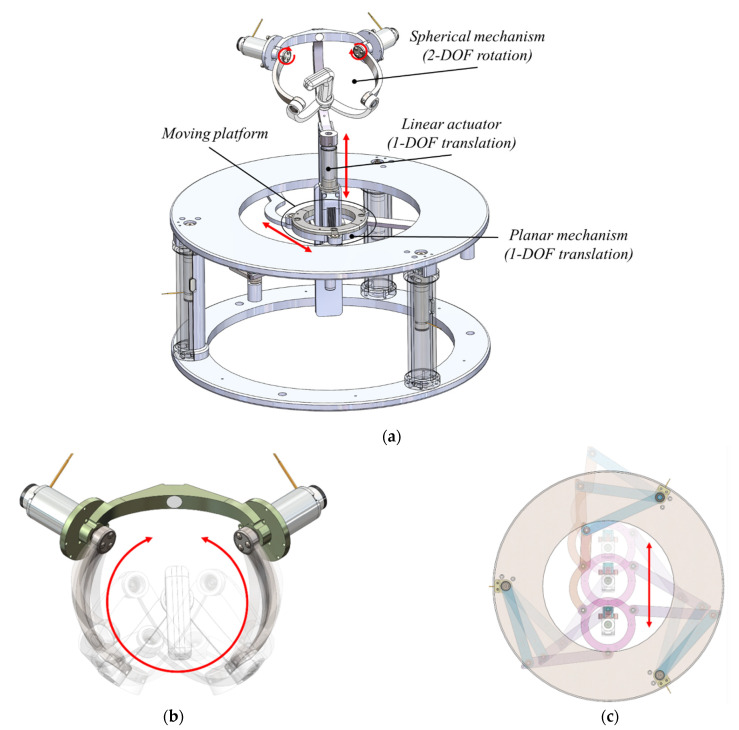
Design of (**a**) the master haptic device and master haptic device matching concept, (**b**) rotational motion, (**c**) translation motion.

The master haptic device, which is an improvement of a design presented in a previous study [25], has been designed as shown in Figure 4a. A planar mechanism is attached to a moving block under the linear actuator, and the spherical mechanism is attached to the top of the linear actuator. The spherical mechanism captures the wrist rotation movement of the physician holding the grip, which corresponds to the rotational motion of the vascular instrument, as shown in Figure 4b. The planar mechanism is a widely known 3-RRR-type parallel mechanism [26,27,28]. It consists of a planar orthogonal motion of two DOFs and a vertical axis rotational motion, allowing a 3-DOF movement. While the physician moves the moving platform on the planar mechanism, the forward and backward translational movement is captured and corresponded to the vascular instruments’ translational motion, as shown in Figure 4c. The linear actuator between the two mechanisms acts as a clutch-like trigger for the movement of the tools. When the physician presses the spherical mechanism downward through the linear actuator, the master–slave teleoperation is connected, and the slave robot motion is locked when released. 

### 2.2. Master–Slave System

#### 2.2.1. Control System

**Figure 5:**
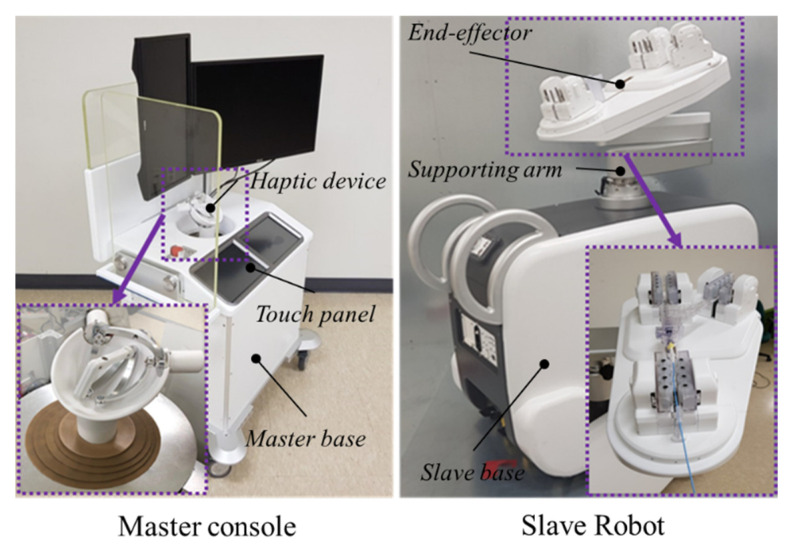
Clinical prototype of the robot system.

Figure 5 shows the prototype of the master–slave system. Most parts of the prototype, excluding specific metal components and the motors, are manufactured from polyester ether ketone. A master console predominantly consists of a haptic device, two touch panels, a master base, and display monitors. Physicians can select the type, size, and speed of the vascular instruments by pushing buttons on the touch panel. The display monitors also show the real-time fluoroscopy image.

**Figure 6:**
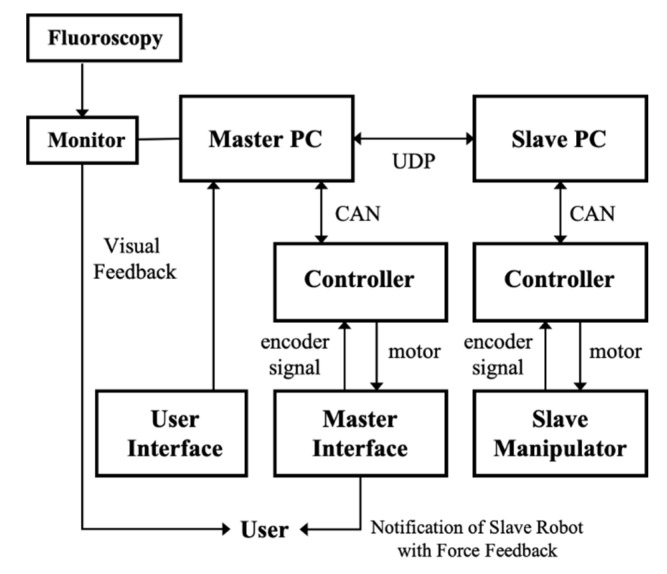
Communication diagram of the master–slave system.

A communication diagram of the master–slave system is shown in Figure 6. Physicians operate the master device on the master console to control the slave end-effector in order to perform the surgery. At the same time, physicians can monitor the fluoroscopy image through the display monitor in real-time to obtain visual feedback. The master haptic device and slave end-effector are actuated using DC motors (Maxon Motor Inc., Sachseln, Switzerland), which are controlled by PCs and motor drivers in the base of the consoles.

The motor controllers are connected to the PC by a controller area network bus set at 500 Hz. The control software was developed in C++, and the graphic user interface on the touch panels was developed using Qt software (The Qt Company, Santa Clara, CA, USA). The communication frequency of the user datagram between the master and slave control systems is 500 Hz.

#### 2.2.2. Motion Model of Vascular Instruments

According to the roller system’s mechanical structure and dimension, the relationships between the axial displacement and rotation angle of vascular instruments and output angles of the corresponding motors can be calculated.

1. Translational motion model: the output angle of the translation motor $\theta_{TR}$ (rad) and axial displacement of vascular instruments, s (mm), satisfies the following relationship:
  (1) $$s=g_{T}\times r\times \theta_{TR}$$
  where $g_{T}$ and *r* (mm) denote the ratio of the bevel gears and radius of the roller, respectively.
2. Rotational motion model: The output angle of the rotation motor and the vertical displacement of the roller modules are $\theta_{RO}$ (rad) and $y_{rm}$ (mm), respectively, which satisfy the following relationship. Then, the rotation angle of the vascular instrument, $\theta_{t}$ (rad), can be obtained by the following:
  (2) $$\theta_{t}=\frac{y_{rm}}{r_{t}}=\frac{g_{R}\times t\times \theta_{RO}}{r_{t}}$$
  where $r_{t}$ (mm) denotes the radius of the vascular instruments; $g_{R}$ and t denote the ratio of bevel gears and ball screw pitch for the elevation of the roller cartridge, respectively.

### 2.3. Compensation for Rotational Motion

#### 2.3.1. Compensation for the Gap of the Roller Cartridge

**Figure 7:**
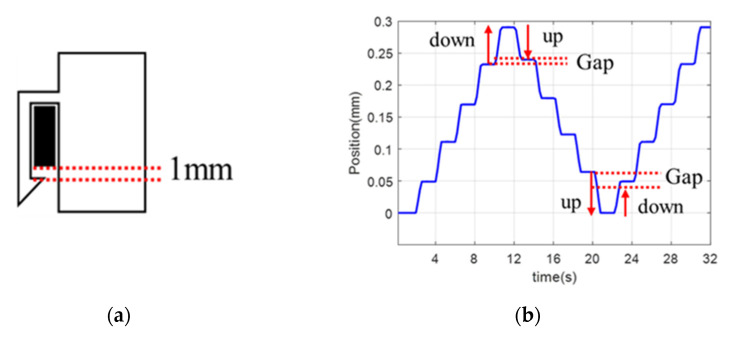
Characteristics of the roller cartridge. (**a**) Gap between the roller cartridge and the bar of the roller module. (**b**) Position of the roller cartridge by pre-compensated control.

As shown in Figure 7a, the gap in the holder is inevitable because the design has a minimal tolerance for assembling and disassembling the disposable roller cartridge from the bar of the roller system. The gap of the roller cartridge is approximately 1 mm. Figure 7b shows an example of backlash in the cartridge’s vertical movement, which will be discussed in Section 4.

Due to this backlash, physicians experience a certain amount of delay in rotational motion when the direction of the motion changes. An experimentally obtained compensation value was used to a resolve the delay.

#### 2.3.2. Haptic Feedback for Rotation Notification

The rotational motion has a limited range due to the limited height of the roller cartridge. The vertical displacement of the roller cartridge during the rotational motion is 25 mm, and the maximum degree of rotation can be between approximately 900° and 1800°, depending on the vascular instrument’s diameter. This appears to be sufficient to rotate the vascular instruments required during surgery.

**Figure 8:**
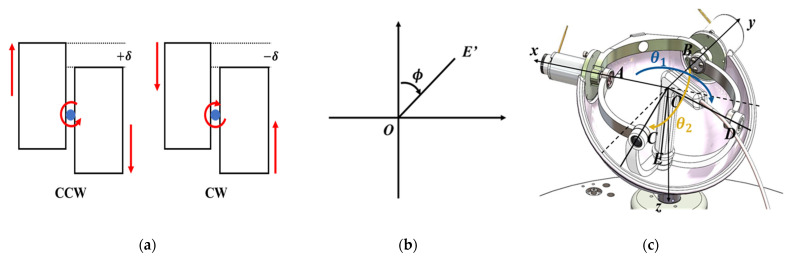
Concept of rotation notification. (**a**) Roller cassettes depending on rotational direction. (**b**) Top view (projection on *x*–*y* plane) of the master grip. (**c**) Isometric view.

For the physician to recognize the rotational motion limit, haptic feedback of the repulsing force is rendered on the master grip when the roller cartridge in the slave end-effector approaches the limit. The haptic feedback is implemented as follows. First, the range of cartridges is set for haptic feedback. As shown in Figure 8a, the difference in height between the two cartridges is set as δ (mm). Then, the movement is performed with the motor’s current control in the spinal mechanical part of the spherical mechanism of the master haptic device, and the force and direction provided to the physician are determined. The inverse kinematics problem of the spherical mechanism is solved to determine the angular displacements of each motor on the mechanism for the given coordinate of *E*. $\bar{\mathrm{AO}}$, $\bar{\mathrm{BO}}$, $\bar{\mathrm{CO}}$, $\bar{\mathrm{DO},}$ and $\bar{\mathrm{EO}}$ intersect at a common point O. $\bar{DO}\;$is in the x–z plane and $\bar{CO}$ is in the y–z plane. Therefore, two angles are defined as $\theta_{1}$ and $\theta_{2}$, as shown in Figure 8c. For the position of the $E^{\prime }$ set as ${\left[x,\;y,\;z\right]}^{T}$ in Figure 8b, the vector of the grip is determined as follows:

(3) $$\bar{OE\prime }=\left[\begin{array}{c} \tan\left(\theta_{2}\right)z\\ \tan\left(\theta_{1}\right)z \end{array}\right],$$

(4) $$\theta_{1}=ATAN2\left(y,z\right)\;\left(\frac{\pi }{2}<\theta_{1}<\frac{3\pi }{2}\right),\;\theta_{2}=ATAN2\left(x,z\right)\;\left(\frac{\pi }{2}<\theta_{2}<\frac{3\pi }{2}\right)\;$$

Next, we determine the amount of force for the haptic feedback according to the roller cartridge range. The current exerted at the position of the roller cartridge, δ, is calculated according to (4). The current drawn is directly proportional to the torque developed by the motor.

(5) $$I_{hf1,2}=\left\{\begin{array}{c} a\times \delta +b\\ 0 \end{array}\right.\begin{array}{c} \left(\delta_{min}\leq \left|\delta \right|\leq \delta_{max}\right)\\ \left(0\leq \left|\delta \right|<\delta_{max}\right) \end{array}$$

where $I_{hf1}$ is the current for the rotation motor on the right of the grip of the master haptic device and $I_{hf2}$ is the current for the rotation motor on the left. The slope and *y*-intercept of the linear function are calculated by (5). The value of each parameter is as follows:

(6) $$\begin{array}{l} a=sgn\times \frac{I_{max}-I_{min}}{\delta_{max}-\delta_{min}},\;b=-a\times \delta_{min}\\ sgn=\left\{\begin{array}{cc} +1 & \left(\theta_{1,2\;current}-\theta_{1,2\;prev}\;\geq 0\right)\\ -1 & \left(\theta_{1,2\;current}-\theta_{1,2\;prev}<0\right) \end{array}\right. \end{array}$$

where $\theta_{1,current}$ and $\theta_{2,current}$ indicate the angles at the current status, and $\theta_{1,prev}$ and $\theta_{2,prev}$ indicate the angles at the previous status.

## 3. Performance Evaluation and Results

Experiments were conducted to evaluate the accuracy and precision of the vascular instruments’ motion when operating the system, and to assess the robot-assisted intervention’s effectiveness.

### 3.1. Compensation Method Evaluation

#### 3.1.1. The Gap in the Roller Cartridge

A laser sensor (HL-G103-S-J, Panasonic, Kadoma City, Japan) was placed vertically on the cartridge, and the vertical displacement of the roller cartridge was measured. An incremental command allows it to move up or down at regular intervals of 0.06 mm, which is the amount required for a 10° rotation of the guidewire. One trial included five increments of descending, ascending, and descending, and a total of 20 trials were performed. The displacement loss value was obtained through the 20 initial experiments without a compensation control. Then, the trials with a compensation control were repeated, changing the rotational direction.

**Figure 9:**
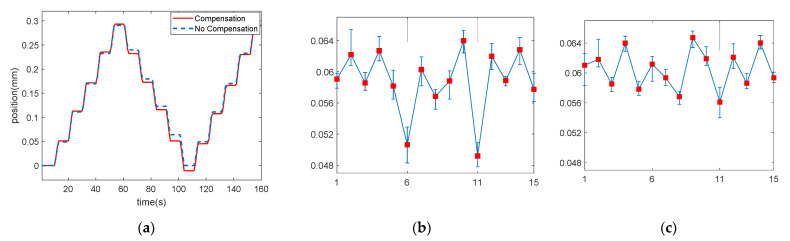
Result of the compensation experiment of the gap of the roller cartridge. (**a**) Position of the roller cartridge from the laser sensor with/without compensation. (**b**) Without compensation. (**c**) With compensation; the data include the median and range of measured errors for 20 trials.

As a result, Figure 9a shows that the proposed compensation method improved the backlash in the roller cartridge’s vertical motion. The accuracy of the system was quantified by measuring the theoretical displacements and the averaged displacements with/without compensation in every trial, and calculating the root mean square error (RMSE). The RMSEs of the displacement with/without the compensation method were 0.0022 mm and 0.0055 mm, respectively. The system was able to follow the desired displacement accurately, reducing the RMSE by 40%.

#### 3.1.2. Haptic Feedback for Rotation Notification

When an physician first rotates the grip of the master haptic device in the clockwise direction, from the initial position for feedback ($-\delta_{min}$), −6.325 mm, to the maximum direction ($-\delta_{max}$), −6.5 mm, of the roller cartridge, we checked whether the torque value of the motor depends on $\theta_{1}$, $\theta_{2}$, and the position of the roller cartridge, in (3) to (6). The two DC motors of the grip have a maximum power of 24 W and maximum continuous torque of 15.3 mNm. Moreover, the torque constant ($K_{t}$) is 18.4 mNm/A. When the physician rotated the grip in the opposite direction, from the initial position for feedback ($\delta_{min}$) of 6.325 mm to the maximum direction ($\delta_{max}$) of 6.5 mm of the roller cartridge, the torques were checked. 

**Figure 10:**
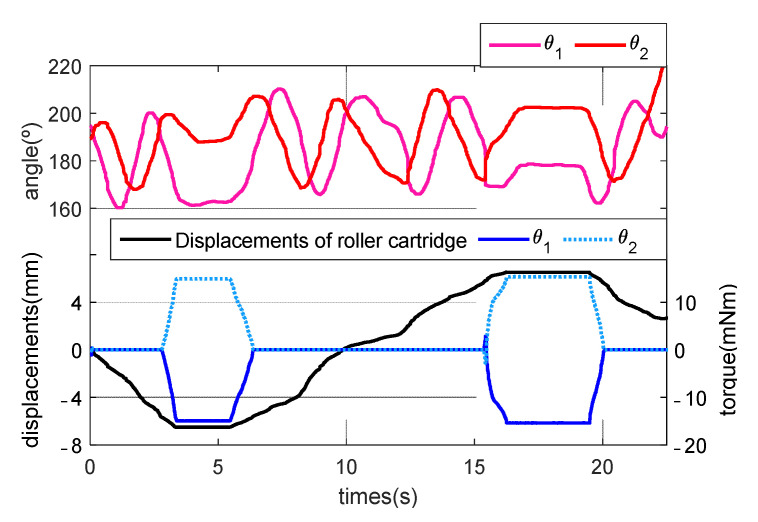
Torques for the haptic feedback depending on the directions of $\theta_{1}$, $\theta_{2}$, and the position of the roller cartridge.

As a result, Figure 10 illustrates the torques for haptic feedback, depending on the directions of $\theta_{1}$ and $\theta_{2}$, and the position of the roller cartridge. When the displacement of the roller cartridge reached from $-\delta_{min}$ to $-\delta_{max}$, the torque of the left motor for $\theta_{1}$ increased linearly and that of the right motor decreased linearly, and the torques of the left and right motors were 15.364 and –15.364 mNm, respectively, at −6.5 mm, for the clockwise rotation. Conversely, the torques of the left and right motors were −15.364 and 15.364 mNm, respectively, at –6.5 mm for the clockwise rotation.

### 3.2. Accuracy Test of the Motion of Vascular Instruments

**Figure 11:**
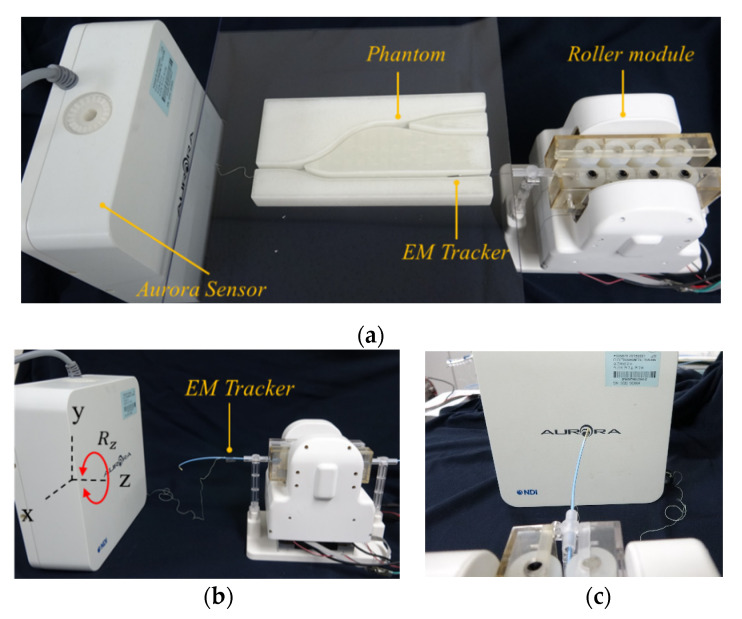
Experimental setup for (**a**) translational motion, (**b**) rotational motion, (**c**) rotational motion in the front view.

We conducted a series of experiments to characterize the accuracy and precision of the modularized system with an active clamping mechanism in the axial and radial directions. The experiment was set up as shown in Figure 11. The vascular instruments used in the experiment were a 5-F catheter, 0.014-inch guidewire, and a balloon catheter (3.5 × 15 mm). The actual displacement and angle of the vascular instruments were measured by using an electromagnetic (EM) tracking sensor with six DOFs (Aurora system, Northern Digital Inc., Waterloo, ON, Canada). The accuracy was evaluated in terms of the absolute error (between the actual displacement/angle of the vascular instruments and the theoretical displacement). The axial and radial precision was evaluated by calculating the standard deviation of the absolute error.

The accuracy and precision in translation were evaluated while the vascular instruments were placed in a 5 mm diameter vascular 2D path model produced by 3D printing, which was used to avoid introducing measurement error owing to the elastic deformation of the vascular instruments. To evaluate the accuracy and precision, the vascular instruments were advanced from 0 to 10, 20, 30, 40, and 50 mm, ten times in succession. An EM sensor was attached at the distal end of the vascular instruments and the spatial coordinates could be provided by the EM tracking system. The actual displacement of the catheter can be calculated by the following:

(7) $$S_{act}=\sqrt{{\left(x_{2}-x_{1}\right)}^{2}+{\left(y_{2}-y_{1}\right)}^{2}+{\left(z_{2}-z_{1}\right)}^{2}\;}$$

where ($x_{1},\;y_{1},\;z_{1}$) is the coordinate of the start point, and ($x_{2},\;y_{2},\;z_{2}$) is the coordinate of the end point.

To evaluate the accuracy and precision of rotational motion, the guiding catheter and guidewire were rotated by the roller module, from 0° to 360°, ten times, and the actual angles were measured. An EM sensor was attached at the position where it begins to flatten from the tip of the vascular instruments. The constant command was sent to the rotation motor repeatedly, to rotate the guiding catheter and guidewire 10 times. The accuracy of the EM tracking system is 0.48 mm RMS per the specification.

As a result, the accuracy and precision of vascular instruments in translation are listed in Table 1, and their accuracy and precision in rotation are listed in Table 2.

### 3.3. Evaluation of the Master–Slave Robotic System

**Figure 12:**
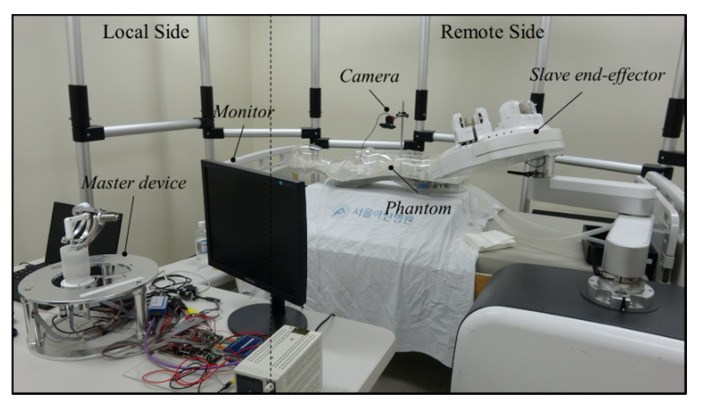
Phantom experimental setup for percutaneous coronary intervention procedure.

To test the transmission accuracy of a guidewire and balloon catheter, the experiment was conducted on a silicon cardiovascular model. The experimental setup, as shown in Figure 12, consisted of two parts. In the remote site, a silicone-based vascular phantom (EndoVascular Evaluator (EVE), FAIN-Biomedical Inc., Japan) was used. The slave end-effector actuated the guidewire and balloon catheter into the EVE model. A camera was mounted immediately beside the EVE model, in order to obtain visual feedback during the experimental process. In the local site, the physician navigated the vascular instruments through the master haptic device to guide the vascular instruments from the femoral artery to several targets in the EVE model. The 5-F guiding catheter placement was performed manually through the brachial artery for arterial access. A 2.5 mm × 15 mm balloon catheter and a 0.014-inch guidewire were loaded into the roller module separately. The image information from the camera was presented on the computer monitor. The guidewire and balloon catheter were controlled by the master–slave robotic system to enter different vascular branches.

**Figure 13:**
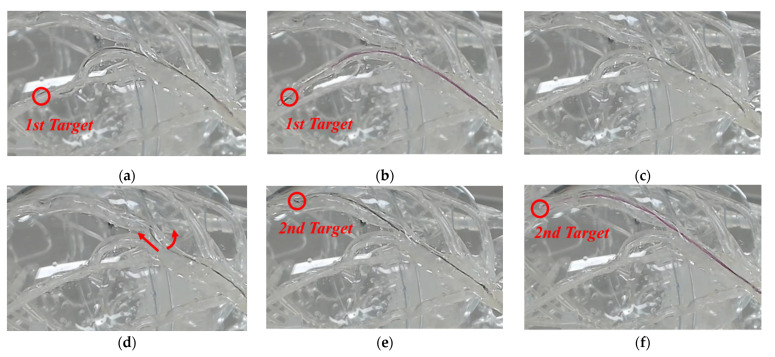
Two-dimensional views of the left coronary artery in the phantom. (**a**) Guidewire is positioned, (**b**) balloon catheter is positioned at the target, (**c**) guidewire is retracted, (**d**) guidewire is advanced, (**e**) it is positioned, (**f**) balloon catheter is positioned at the second target.

**Figure 14:**
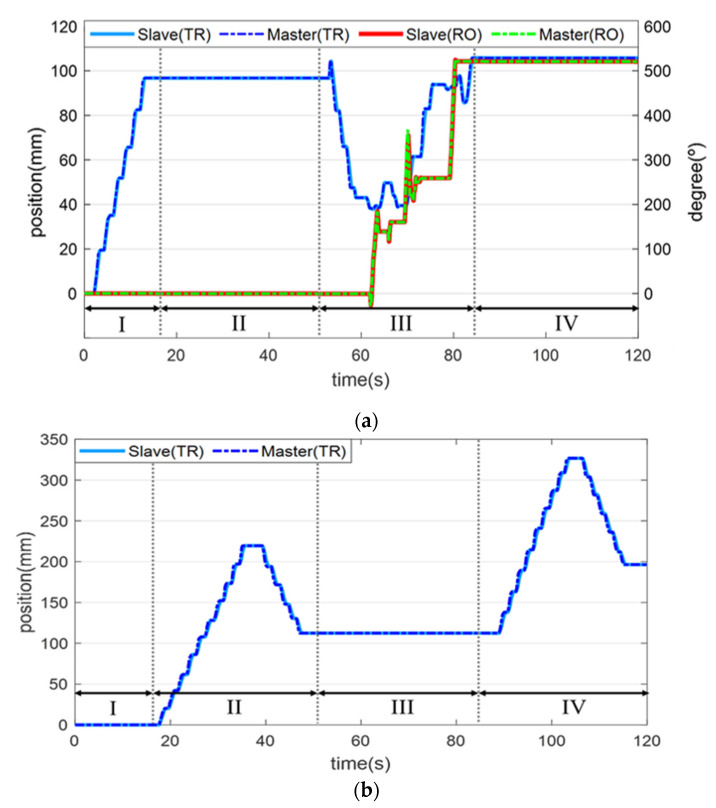
Translation and rotation information of the (**a**) guidewire and (**b**) balloon catheter when they are navigated to the target points.

Figure 13 shows that the guidewire and balloon catheter were located in the two targets. Figure 13a shows that the guidewire was advanced and positioned at the first target, and Figure 13b illustrates that the balloon catheter was advanced through the guidewire to the target point, and positioned fully at the first target. As shown in Figure 13c,d, the physician worked on the master device to operate the slave end-effector, to achieve simultaneous translational and rotational motions of the guidewire, and the guidewire was advanced to a new branch quickly and accurately. In Figure 13e,f, it can be observed that the guidewire advanced and reached the second target. Then, the balloon catheter was also advanced through the guidewire and positioned at the second target. The experiment confirmed that the guidewire and balloon catheter can be smoothly moved and precisely positioned using the proposed system. Figure 14 illustrates the translational and rotational motion trajectories of the guidewire and balloon catheter in the master and slave systems in the phantom experiment. In stage 1, the guidewire was inserted into the first target for translational motion, and in stage 2, the balloon catheter was inserted into the first target. Then, in stage 3, the guidewire was simultaneously advanced and rotated to move to the other branch, and it reached the second target. In stage 4, the balloon catheter was inserted into the second target.

### 3.4. Animal Experiment

**Figure 15:**
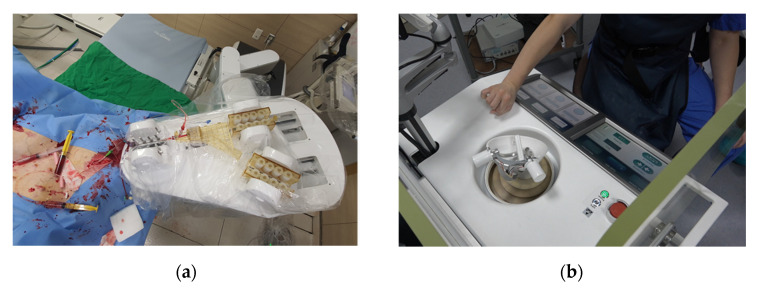
Setup of the animal experiment. (**a**) The slave robot beside the operation table and (**b**) the physician at the master console.

An animal experiment, using a porcine model, was performed to verify the proposed system’s functionality and safety under an IACUC (Institutional Animal Care and Use Committee) approval of the Osong Medical Innovation Foundation, Republic of Korea (KBIO-IACUC-2019-105-1), as shown in Figure 15. The guiding catheter was manually inserted and engaged in the coronary ostium in a 60 kg female pig. The physician operated the master console to remotely control the slave end-effector for the translation and rotation of the guidewire under angiography image guidance. The robotic system inserted a guidewire from the entrance of the coronary artery to the targets in the right coronary artery. 

**Figure 16:**
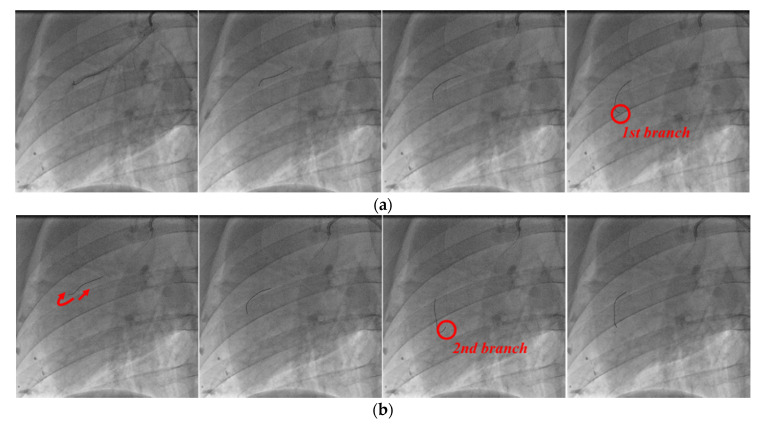

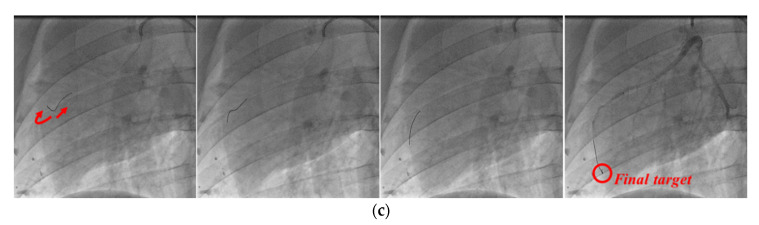
Angiographic views of the right coronary artery of the pig. Process of inserting guidewire into (**a**) first branch, (**b**) second branch, and (**c**) final target.

The angiography images of each stage in the process of guidewire advancement are shown in Figure 16. The guidewire was advanced, and it reached the first branch point, as shown in Figure 16a. Then, the guidewire was withdrawn from the first branch by moving it backward and rotating it, and it was advanced again to reach the second branch, as shown in Figure 16b. Finally, the guidewire was retracted and rotated again to exit from the second branch point, and then it was advanced to successfully reach the final target location, as shown in Figure 16c. In postoperative observation, the physician confirmed that there were no complications, such as perforation or bleeding.

### 3.5. Clinical Trial

**Figure 17:**
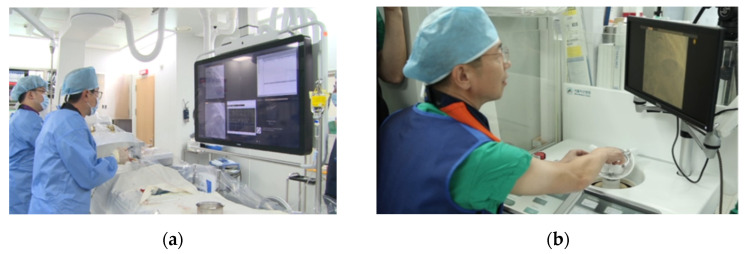
Setup of the clinical trial. (**a**) The slave robot and the patients in the operation room are shown. (**b**) The physician is located in the corner of the angiography room with the master console, behind a radiation-shielded glass wall.

A single-center, single-group clinical trial was conducted on two patients requiring coronary angiography at Asan Medical Center, Seoul, Republic of Korea (Asan Medical Center Institutional Review Board Registration Number: S2018-2377-0002, Korean Ministry of Drug and Food Safety approval: 20190064902), as shown in Figure 17. The clinical trial procedure was conducted as follows:

1. Setup of robot was prepared, such as sterile drapes and loading detachable sterilized rollers.
2. Arterial access and guiding catheter placement of coronary ostium were performed manually.
3. After the guiding catheter was completely located in the coronary artery, using the manual robot arm in the slave robot, the slave end-effector was positioned beside the bed on which the patient was lying, and the master console was located far from the patient. A guidewire was loaded by a tableside assistant into appropriate roller modules, which served as the sterile interface between the slave end-effector and the patient.
4. The physician sat in front of the master console, behind a radiation-shielded wall, and used the master haptic device and touchscreen controls to advance, retract, rotate, and deploy the devices as required.
5. A guidewire was inserted into the target vessel site using a robotic system, through a guiding catheter inserted manually.
6. When the guidewire was positioned at the desired location, coronary angiography was completed.
7. After 4 h of bed stability, patients were monitored for bleeding complications, and at 24 h after the procedure, patients were checked for the occurrence of an abnormal event/severe abnormal event.

**Figure 18:**
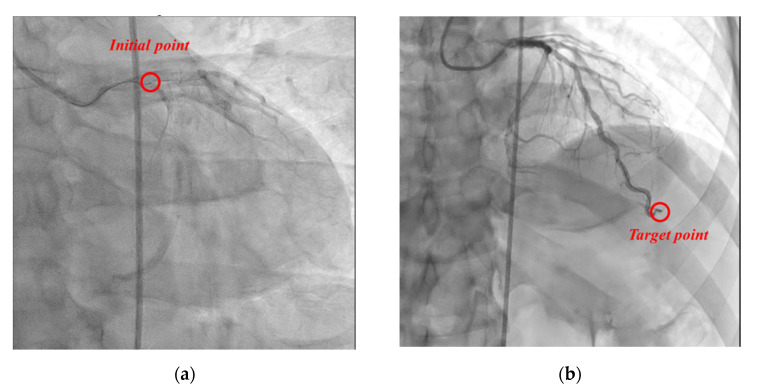
Angiographic views of the left coronary artery of patient 1. (**a**) The guidewire was located at the entrance of coronary. (**b**) The guidewire was located at target point.

For efficacy evaluation, in the coronary angiography of the two subjects participating in this clinical trial, the guidewire was transferred to the target site of the coronary artery (subject 1: “left anterior descending artery”, subject 2: “right coronary artery”) using the proposed master–slave robotic system, without manual manipulation, as shown in Figure 18. At this time, there was no damage or dissection of the distal intima (“Coronary artery dissection—The National Heart, Lung and Blood Institute classification” criteria, evaluated as type A). Accordingly, coronary angiography using the proposed master–slave robotic system of the two subjects registered in this clinical trial was evaluated as a “technological success”.

## 4. Discussion

A master–slave robotic system with roller cartridge-based modules for robotic cardiovascular intervention has been developed for clinical application. Preliminary experiments were conducted to evaluate the performance of the roller cartridge-based modularized robotic system. Specific problems associated with the roller cartridges were adequately alleviated by compensation control and a force rendering method using the master haptic device. Through the experiment using the phantom, we checked the functionality and safety of the master–slave robotic system. It was confirmed that no significant problems were identified in the master–slave system, and the positioning precision and accuracy of the guidewire and balloon catheter were also sufficiently high. It was also shown that the combination of translation, rotation, and their simultaneous motion enables vascular instruments to navigate to the desired location in the vascular branches. The in vivo animal experiment confirmed that navigating a guidewire in several branches worked smoothly as the physician manipulated the master haptic device. In the clinical trial, the initial verification and validity of the prototype of the master–slave robotic system was confirmed by navigating the guidewire to the target location completely. Further in vivo tests should be performed to assess the clinical efficacy of the system.

Maor et al. presented the limitations of an existing percutaneous coronary intervention (PCI) robot, such as the absence of tactile feedback and limited applicability to various complex vascular intervention procedures [29]. The system presented in this paper is similar to the Corpath system in its purpose and instrumentation motion. Although a haptic device has been implemented in the master console, the utilization was only for a virtual wall in the rotation operation. Further development and evaluation are needed and are underway to appropriately show the usefulness of the haptic device that is designed to capture and interact with the physician’s positional and rotational motion via the 6-DOF mechanism. The haptic device’s prominent role seems to be more for the haptic rendering to augment the virtual structure and sensation to facilitate the physician’s situation awareness and instrumentation maneuver than repulsive force feedback, which is limited by various attenuation factors in PCI procedures. Some physicians who tested the system in the usability viewpoint commented that PVI (peripheral vascular intervention) procedures might better utilize the force feedback.

The force feedback can be helpful in the manipulation of guidewires in PCI or PVI procedures. However, guidewires have soft and flexible ends for safer maneuver in the blood vessel. The repulsive force is largely attenuated due to the tip property and various frictions over the vessel path. In the cardiac ablation procedures, the contact force of the ablation catheter is essential and can be sensed by various methods, such as direct measurement using sensor structures embedded in the catheter, or indirect estimation using image analysis of the catheter body shape deformation in fluoroscopy. It is difficult to sense or estimate the repulsive force over the whole range in the case of the guidewire in the PCI procedure. Although relatively large repulsion, such as hitting the obstruction or friction during passing through an abrupt curve in the vessel, can be sensed at the proximal part of the instrument, physicians’ experience and opinions vary. The methods for the repulsive force measurement and direct force feedback need further study, and this is under experiment by many researchers, including the authors.

Unlike marvelous human finger motion, artificial mechanical systems should be passive locking mechanisms with fixed grabbing forces, or active motorized mechanisms with more complex and bulky structures. As the complexity of the PCI procedure increases, various instruments, with different diameters, internal structures, and stiffness of the enclosing material, are engaged. The active clamping mechanism in our system is to facilitate those complex PCI procedures better. The integrated cassette configuration of the Corpath systems’ disposable part, enclosing all parts in one closed housing, has the advantage of relatively easy and fast installation, among other features. In comparison, our system modularized and separated all the disposable parts without closed housing. As a result, the volume and surface area of the sterile parts are relatively smaller, and the reduced complexity in the structure favors reliability. In an emergency, manual conversion can be more easily and quickly performed by simply lifting the instruments minimizing unwanted motion inside the patient, since there is no enclosing structure.

There is a need for a method to solve various nonlinear factors caused by interactions between surgical environmental factors, instruments, and robots. Machine learning-based control methods, such as reinforcement learning, could be a plausible approach. In addition, a new slave end-effector mechanism for complex PCI and control methods, to ensure autonomy in robotic procedures and perform safer tasks in less time, is under development.

## 5. Conclusions

In this study, a novel master–slave robotic system was developed. Pre-clinical in vitro and in vivo tests showed that the slave end-effector could control the translational and rotational motions of the vascular instruments with satisfactory performance. The clinical trial proved the essential safety and performance of the system to effectively assist the physician in vascular interventions.

## Figures and Tables

**Table 1:** Accuracy and precision of translational motion of vascular instruments.

Tool	Theoretical Displacement (mm)	Averaged Actual Displacement (mm)	Mean Error (mm)	Standard Deviation (mm)
Guiding Catheter	10	9.38	–0.623	0.882
	20	19.54	–0.46	0.987
	30	29.01	–0.736	1.024
	40	39.07	–0.93	0.43
	50	50.75	0.75	0.12
Guidewire	10	10.71	0.71	0.39
	20	20.64	0.64	0.63
	30	29.9	0.11	0.54
	40	40.86	0.86	0.74
	50	50.24	0.24	0.74
Balloon Catheter	10	10.31	0.31	0.04
	20	20.07	0.07	0.03
	30	30.11	0.11	0.08
	40	40.57	0.57	0.42
	50	50.13	0.13	0.37

**Table 2:** Accuracy and precision of rotational motion of vascular instruments.

Tool	Theoretical Angle (°)	Averaged Actual Angle (°)	Mean Error (°)	Standard Deviation (°)
Guiding Catheter	360	363.29	3.29	5.48
Guidewire	360	361.93	1.93	2.50

## Data Availability

Not applicable.
